# Predicting Eye and Hair Color in a Turkish Population Using the HIrisPlex System

**DOI:** 10.3390/genes13112094

**Published:** 2022-11-11

**Authors:** Ilksen Sari O, Sumeyye Zulal Simsek, Gonul Filoglu, Ozlem Bulbul

**Affiliations:** 1 Institute of Forensic Sciences and Legal Medicine, Istanbul University-Cerrahpasa, 34500 Istanbul, Turkey; 2 Department of Medical Services and Techniques, Vocational School of Health Services, Istanbul Gelisim University, 34310 Istanbul, Turkey

**Keywords:** HIrisPlex, population differences, prediction accuracy, forensic DNA phenotyping

## Abstract

Forensic DNA Phenotyping (FDP) can reveal the appearance of an unknown individual by predicting the ancestry, phenotype (i.e., hair, eye, skin color), and age from DNA obtained at the crime scene. The HIrisPlex system has been developed to simultaneously predict eye and hair color. However, the prediction accuracy of the system needs to be assessed for the tested population before implementing FDP in casework. In this study, we evaluated the performance of the HIrisPlex system on 149 individuals from the Turkish population. We applied the single-based extension (SNaPshot chemistry) method and used the HIrisPlex online tool to test the prediction of the eye and hair colors. The accuracy of the HIrisPlex system was assessed through the calculation of the area under the receiver characteristic operating curves (AUC), sensitivity, specificity, positive predictive value (PPV), and negative predictive value (NPV). The results showed that the proposed method successfully predicted the eye and hair color, especially for blue (100%) and brown (95.60%) eye and black (95.23) and brown (98.94) hair colors. As observed in previous studies, the system failed to predict intermediate eye color, representing 25% in our cohort. The majority of incorrect predictions were observed for blond hair color (40.7%). Previous HIrisPlex studies have also noted difficulties with these phenotypes. Our study shows that the HIrisPlex system can be applied to forensic casework in Turkey with careful interpretation of the data, particularly intermediate eye color and blond hair color.

## 1. Introduction

Genome-wide association studies (GWASs) have been a powerful tool for unraveling the molecular genetic basis underlying natural human phenotypic variations. Through GWAS, many single nucleotide polymorphisms (SNPs) that are involved in human phenotypic variations (i.e., eye, hair, and skin color) have been identified [1,2,3]. For over two decades, forensic scientists been benefitted from the GWASs by producing new forensic clues (prediction of the eye, hair color, etc.) for criminal investigations [3,4,5]. Forensic DNA Phenotyping (FDP) is defined as an investigative tool through which biogeographic ancestry, externally visible characteristics (EVCs), and age are predicted by using the DNA obtained at a crime scene [4,5]. In standard forensic DNA profiling, short tandem repeats (STRs) are used to identify biological evidence collected from crime scenes. However, STR profiling has limitations because of the nature of the comparison-based analysis method. In cases where there is no suspect or the DNA profile obtained from the crime scene is not recorded in forensic DNA databases, STR profiling is of limited value in the investigations. In such situations, FDP can help the police investigation to narrow down the potential suspect groups by determining features of the perpetrator’s physical appearance. FDP can also be applied to other cases such as in the identification of mass disaster victims and missing persons [5,6,7,8].

The first FDP study started with the identification of the MC1R (melanocortin 1 receptor) gene associated with the red hair and freckles’ variation in normal populations [9]. More than 200 SNPs were found to be highly polymorphic predominantly in Europeans. Grimes et al. developed a small panel consisting of 12 MC1R SNPs to accurately assign individuals with red hair color. However, red hair color prediction had limited application in forensics due to its low prevalence (1–2%) globally [9]. The first comprehensive FDP study was conducted by Liu et al. [10] by selecting the most informative SNPs for eye color and developing statistical models that accurately predicted eye color. Afterward, Walsh et al. [11] used a small subset of this study and developed the IrisPlex system to predict blue and brown eye color. The IrisPlex panel includes six SNPs from six genes (rs12913832 in HERC2, rs1800407 in OCA2, rs12896399 in SLC24A4, rs16891982 in SLC45A2, rs1393350 in TYR, and rs12203592 in IRF4) and can be genotyped using SNaPshot chemistry and capillary electrophoresis. The developed prediction model was based on a multinomial logistic regression (MLR) that calculates the prediction probabilities for blue, brown, or intermediate (green-hazel) eye color using genotype and phenotype information from European databases [11]. This panel was subsequently tested for various populations including admixed and intermediate populations and showed similar accuracies with Europeans [12,13,14]. 

In the last years, the HIrisPlex system, which includes a single multiplex genotyping assay for 24 SNPs, was developed by the same research group by extending the eye color panel with additional 18 SNPs that are associated with hair color variations [15]. The panel consists of a total of 24 SNPs from 11 genes (10 SNPs and one InDel from MC1R gene, two SNPs from SLC45A2, two SNPs from TYR, two SNPs from SLC24A4, one from KITLG, one from EXOC2, one from IRF4, one from OCA2, one from HERC2, one from ASIP/PIGU, and one from TYRP1). The model-based prediction could be conducted for eye color with six SNPs and hair color with all SNPs except two (rs1393350 in TYR and rs12896399 in SLC24A4). The HIrisPlex prediction model was developed by using European populations [15]. The HIrisPlex system has also been tested on the HGDP-CEPH sample set. The researchers concluded that the performance of the HIrisPlex systems was independent of the biogeographic ancestry. However, this dataset does not provide phenotype information on the samples [16]. Before applying this panel to forensic cases, its precision for non-European populations should be tested. Therefore, the main objective of this study is to estimate the precision of the HIrisPlex panel on the Turkish population.

## 2. Materials and Methods

### 2.1. Sample Collection

Buccal swabs were taken from 149 unrelated volunteers (72 males, 77 females) living in Istanbul, Turkey. All individuals were randomly selected, except for four red-haired individuals who were invited because red hair is not widespread in Turkey. The samples were also selected according to their birthplace and the parents’ birthplace to reflect seven geographical regions (Mediterranean Region, Black Sea Region, Aegean Region, Marmara Region, Central Anatolia Region, Eastern Anatolia Region, Southeastern Anatolia Region) of Turkey. All volunteers were asked to complete a questionnaire that included basic information such as gender, ancestry, age (between 18–69, on average 31.30), and data concerning eye and hair pigmentation phenotypes. The volunteers’ statements on their hair and eye colors were recorded on consent forms. The photos were taken in portrait mode with equal light intensity using a Nikon D5100, 18–55 mm lens at an equal distance. Two independent researchers assigned each volunteer’s eye and hair color into categories (for eye color: blue, intermediate (green/hazel), and brown; for hair color: red, blond, brown, and black), according to the phenotyping regimes applied by Walsh et al. [11,15,16]. The HIrisPlex predictions were compared to the actual phenotypes of volunteers based on these data. During the course of our study, we noted volunteers with hair color changes from childhood to adulthood.

Eye colors were classified into three categories: brown (61.07%), intermediate (25.50%), and blue (13.42%). Hair colors were classified into four main categories: blond (19.45%), brown (63.75%), black (14.7%), and red (2.68%).

### 2.2. DNA Samples and Genotyping

Genomic DNA was purified from buccal swabs using the QIAamp DNA Mini Kit (Qiagen, Hilden, Germany). The amount of extracted DNA was measured with the Qubit^®^ fluorometer using the Qubit^TM^ dsDNA HS Assay Kit (Invitrogen, Thermo Fisher Scientific, Waltham, MA, USA), according to the manufacturer’s protocols. 

We used the HIrisPlex panel, a 24-plex assay (23 SNPs and 1 InDel) that has been developed over the years for predicting eye and hair color [15]. HIrisPlex SNPs were genotyped according to the protocol described by Walsh et al. 2014 [15]. Multiplex PCR was set up with a total volume of 10 μL and contained 4 μL of Qiagen Multiplex Master Mix, 2.84 μL of PCR primer mix (0.4–0.5 μM), 0.5–1 ng DNA, and 0.16 μL nuclease-free water. PCR was performed using a SimpliAmp™ Thermal Cycler Applied Biosystems (Thermo Fisher Scientific) with the following program: denaturation at 95 °C for 10 min, then 33 cycles of 95 °C for 30 s, 61 °C for 30 s, and final extension at 61 °C for 5 min. Excess primers and dNTPs were removed by the addition of 0.25 μL Exo, 0.75 shrimp alkaline phosphatase (SAP) to 2.5 μL PCR product, incubation at 37 °C for 90 min, followed by enzyme inactivation at 85 °C for 15 min. Single base extension reactions (SBE) were carried out in 5 μL volumes containing 1 μL of SNaPshot^®^ reaction mix (Thermo Fischer Scientific), 1.61 μL of SBE primer mix (0.1 μM), 0.39 μL nuclease-free water, and 2 μL of purified DNA under the following conditions: 30 cycles of 96 °C for 10 s, 50 °C for 5 s, and 60 °C for 30 s. Excess nucleotides were removed by the addition of 1 μL SAP to the total volume of extension products, and incubation at 37 °C for 80 min, and then by enzyme inactivation at 85 °C for 15 min. Capillary electrophoresis was performed using an ABI PRISM 3130 Genetic Analyzer (Thermo Fischer Scientific). The matrix standard DS-02 and POP-4 polymers were used on the AB PRISM 3130 Genetic Analyzer with filter set E5 to process the data. Genotypes were generated with GeneMapper v4.0 (Thermo Fischer Scientific) or Peak Scanner Software v2.0 (Thermo Fischer Scientific).

### 2.3. Statistical Analysis

Phenotype predictions were performed using the online HIrisPlex tool (https://hirisplex.erasmusmc.nl/, accessed on 14 November 2021). The model calculates the prediction probabilities for the eye (blue, brown, and intermediate) and the hair (black, brown, blond, and red) colors using the multinomial logistic regression (MLR) model. For eye color, the threshold was set as 0.7 as suggested by Walsh et al. [11]. Hair color was classified according to the 0.5 probability threshold determined by Walsh et al. [16,17]. The predicted eye and hair phenotypes were then compared with the true phenotypes.

The overall prediction accuracy of the model for each phenotypic character was assessed by calculating the area under the curve (AUC) and the receiver operating characteristic (ROC) curves using a script written in Python 3. Moreover, the sensitivity, specificity, positive predictive value (PPV), and negative predictive value (NPV) of the model were calculated according to Liu et al. [10]. The sensitivity was determined as the percentage of the truly predicted color type among all observed color types. The specificity was defined as the percentage of truly predicted non-color type among the observed non-color type [10].

We also compared the allele frequencies of the HIrisPlex SNPs with the European population data from 1000 Genomes Phase 3 (Utah residents with ancestry from Northern and Western Europe [CEU], Finnish [FIN], British from England and Scotland [GBR], Iberians from Spain [IBS], and Toscani in Italy [TSI]) to explore the ancestry differences between populations. SNP frequencies of the Turkish population were calculated by gene counting.

## 3. Results and Discussion

In this study, we evaluated the performance of the HIrisPlex system for the Turkish population. Before testing the population samples, we completed an internal validation study according to the validation guidelines for forensic DNA analysis methods of the Scientific Working Group on DNA Analysis Methods (SWGDAM) [18]. In the validation of the study, we determined that the sensitivity of the panel required a minimum of 0.25 ng DNA input [18]. In the present study, we successfully genotyped our 149 samples using the single base extension (SBE) approach and SNaPshot chemistry. In a few samples (N = 8), the N29InsA InDel variant’s peak heights were too low to detect. This InDel variant was reported as the lowest peak in the assay and it plays a minor role in predicting red hair color. Thus, the missing data for N29InsA InDel did not affect the red hair prediction [16]. In the samples with no N29InsA data (none of them have red hair color), we applied the prediction model without re-genotyping the samples, and the predictions were accurate, as expected. However, when a hint of red hair color is observed in a case, the sample should be repeated to obtain this variant for accurate red hair color prediction.

To determine the effect of ancestry on the phenotype prediction, we compared the Turkish population allele frequencies with the European population from the 1000 Genomes Project Phase 3 data. The allele frequencies of our population were very similar to that of the overall European 1000 Genomes population data except for SNP rs683, rs16891982, and rs12913832 (Supplementary Table S1). rs683 in the TYRP1 gene is associated with eye and hair color [19]. Therefore, this SNP was added to the HIrisPlex panel. When the TT genotype is present at rs683, it helps differentiate European from non-European populations. The T allele frequency was lower in the Turkish population (0.33) than in all of the European populations (0.63). rs1689198 in SLC45A2 was associated with hair color and skin pigmentation. The rs16891982 (C) variant increases the likelihood of having darker hair color [20,21]. The allele frequency of the C allele was observed as 0.26 in the Turkish population. Although the allele frequency of the C allele was 0.06 in the overall European population, the Mediterranean IBS population showed the highest allele frequency among Europeans (0.18) (Supplementary Table S1). rs12913832 in HERC2, a well-known SNP near the OCA2 gene, functionally linked to blue or brown eye color due to a lowering of promoter activity of the OCA2 gene. The GG genotype is strongly associated with blue eye color and European ancestry [22]. In our data, the frequency of the G allele was calculated as 0.43. The overall European data showed a higher frequency (0.64). The Mediterranean-Southern European populations (IBS and TSI) had similar frequencies to the Turkish population (0.32 and 0.42, respectively) (Supplementary Table S1).

### 3.1. Eye Color Prediction

**Figure 1:**
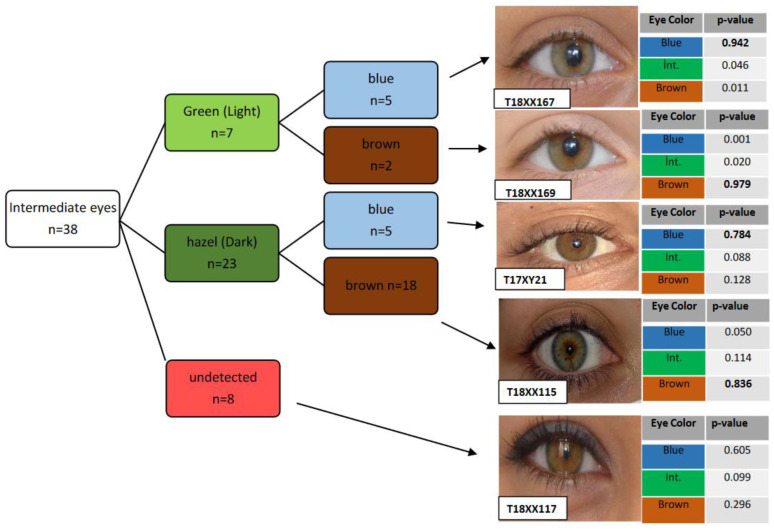
Distribution of the intermediate eye color prediction results. Bold *p*-values indicated the highest *p*-value for the predicted eye color.

The actual eye color of the individuals and their predictions were classified into three categories: blue, intermediate, and brown. We preferred a conservative approach and applied a threshold value (*p* > 0.7) for eye color predictions. After applying the threshold value, we observed the prediction success for blue eye of 100% (*n* = 20) and brown eye of 95.60% (*n* = 91) in the Turkish population samples (Table 1). The incorrect assignment of the intermediate eye phenotypes was 78.94% (30 out of 38) while 21.05% (eight out of 38) of the intermediate eye phenotypes were under the 0.7 threshold for prediction. Most of the intermediate eye color phenotypes (52.63%) were predicted as brown, and approximately a quarter (26.31%) of them were classified as blue with the HIrisPlex MLR model (Table 1). We also classified intermediate eye colors as light and dark colors. The detailed classification of the intermediate eye colors indicates that most of the “light intermediate eyes” were predicted as blue, and similarly, most of the “dark intermediate eyes” were predicted as brown (Figure 1). Similar results were observed in Italian population samples [14].

The eye color model was developed to predict only blue and brown eye colors. The prediction results for intermediate eye color individuals were, however, mainly false positives, either blue or brown [15]. If the eye color of a suspect was predicted as blue or brown, the possibility of the real eye color of the sample could be intermediate (hazel green or green). Intermediate eye color prediction is difficult, and this can be explained by its complex genetic structure. Some studies have focused on understanding the genetic structure of the intermediate eye color and finding new SNPs [23,24]. Kukla-Bartoszek et al. developed an advanced machine learning-based prediction model that increased the sensitivity of the intermediate eye color prediction by up to 39% [24]. However, current assays and prediction models cannot predict the intermediate eye color with high accuracy.

The prediction accuracy AUC values for each eye color were 0.66 for blue, 0.88 for brown, and 0.59 for intermediate (Table 1). The obtained sensitivity values were very high for the brown (95.6%) and blue (100%) eye colors. The sensitivity was not calculated for the intermediate eye color prediction due to no correct classification of the analyzed intermediate-eyed individuals.

The specificity values were very high for blue (92.24%) and intermediate (100%) eye colors while the specificity was lower for brown (65.51%) eye color. This can be explained by the high number of intermediate-eye-colored individuals who were classified as mostly brown (Table 1 and Figure 1). We also calculated the positive predictive value (PPV) and negative predictive value (NPV) according to Liu et al. [10]. PPV and NPV values were 66.66% and 100% for blue, 81.30% and 90.47% for brown, respectively. The PPV value was not computed for the intermediate eye color due to no correct prediction, and the NPV value was 74.49% (Table 1).

Liu et al. (2009), who developed the prediction model, tested the IrisPlex SNPs on 6168 Dutch European populations. The accuracy of the eye color prediction was over 90% for blue and brown eye colors [10]. In our previous study, based on only the IrisPlex assay, we observed a high accuracy of 95.77% and 100% for brown eye colors on 100 Turkish individuals [13]. Salvoro et al. (2019) tested four different eye color prediction models (IrisPlex, Ruiz, Allwood, and Hart models) for IrisPlex eye color SNPs in a sample of 296 Italians. They applied different thresholds (0.7, none, and 5:3 top probability ratio) for the IrisPlex model eye color assignments. The sensitivity was highest (99% for brown and 98% for blue at 0.7 thresholds). Among the overall performance of the four eye color prediction tested models, when the IrisPlex model was applied, the misclassification was the lowest frequency (17%), while the number of inconclusive results was the highest (18%) [14].

The success of the blue and brown eye color predictions of the HIrisPlex (or IrisPlex) system has been proven by different researchers who tested different population samples. Similarly, a high percentage of false predictions for intermediate eye color have been reported [12,13,25,26,27,28]. Our results of the eye color predictions were in agreement with previous studies.

### 3.2. Hair Color Prediction

The actual hair colors of the individuals were divided into four independent classes: red, blond, brown, and black hair. The prediction distributions were made according to the highest observed *p*-value from the four phenotype classes (black, brown, blond, and red). Then, within the highest categories, the dark or light *p*-values for color shade were considered for the final phenotype prediction [15,16]. The prediction success of hair colors was 95.23% (*n* = 21) for black, 96.90% (*n* = 97) for brown, 59.25% (*n* = 27) for blond, and 75% (*n* = 4) for red, respectively (Table 2). A total of 89.26% of the samples correctly predicted their true hair colors. The most inaccurate prediction was blond hair being brown with 40.74% (11 out of 27) (Table 2). All black hair-colored individuals were correctly classified, except for one individual who was assigned to have brown hair color. Three out of 97 brown hair-colored individuals were incorrectly predicted in which two of them were assigned to have black and one of them was assigned to have blond hair color. None of the black, brown, or blond hair-colored individuals were classified as red. However, one of our red hair-colored individuals (one out of four) was incorrectly predicted to have blond hair (Table 2).

The hair prediction accuracy AUC values were observed as high for black (0.96), brown (0.89), blond (0.90), and lower for red (0.55) (Table 2). The obtained sensitivity values were high for the black (95.23%) and brown (96.90%) hair colors. The sensitivity values for blond and red hair were lower (59.25% and 75.00%, respectively). The PPV and NPV values were high for all hair colors (0.88–100) (Table 2).

**Figure 2:**
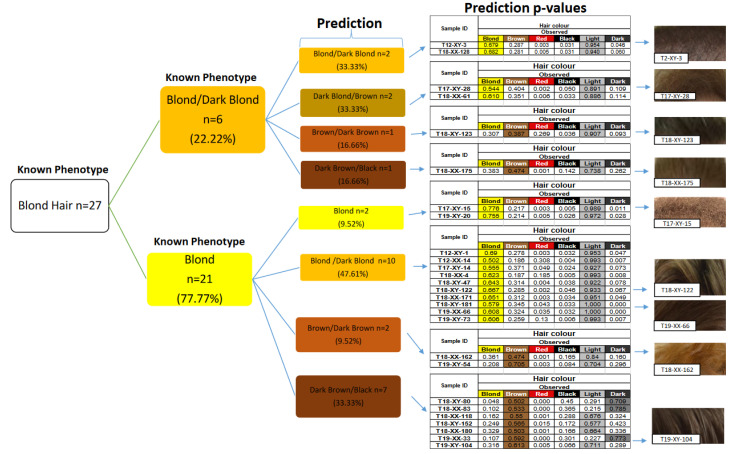
Distribution of the blond hair colored individuals (N = 27) and their prediction results.

The lowest sensitivity value was obtained for blond hair as a result of the highest proportion of incorrect predictions (Table 2 and Figure 2). The blond hair predicted phenotypes were divided into three classes: blond, blond/dark blond, dark blond/brown, and depending on the light *p*-value as described by Walsh et al. [16]. In our population, almost half of the blond-haired individuals were correctly predicted to have blond or blond/dark blond (simply called blond). The highest *p*-values were detected in the brown class for 11 individuals whose actual hair color was blond or dark blond. Therefore, these individuals were incorrectly predicted to have brown hair (Figure 2). For example, the sample T19-XY-104 has blond hair. However, the hair phenotype prediction was brown because of the highest *p*-value in the brown category (brown *p*-value 0.613, light *p*-value 0.711) among the other phenotype categories (blond, red, and black).

The specificity was highest for the red, black, and blond hair colors (100%, 98.43%, and 98.36%, respectively). The specificity of the brown hair color was lower (76.92) due to incorrectly predicted individuals (mostly blonds) as brown. Hence, in an investigation, if the unknown sample is predicted as brown, the true hair color is highly possible to be brown, but also possible to be blond and maybe black hair color. Therefore, the analyst should consider that the suspect could have hair color varying from dark blond to black.

The HIrisPlex panel includes 22 SNPs for hair color prediction [16]. The HIrisPlex assay was tested on >1500 individuals from Western (Irish), Eastern (Polish), and Southern (Greek) parts of Europe to assess the power of the hair color prediction of the panel. HIrisPlex hair color prediction model accuracies were 0.93 for red, 0.87 for black, 0.82 for brown, and 0.81 for blond hair color [16]. Our results are similar to these findings except for the red (0.75) and blond hair (0.59) color prediction accuracy. This difference in red hair color can largely be attributed to the small sample size in our study (only four individuals) than the one used by HIrisPlex. In fact, the frequency of red-haired individuals is very low in the Turkish population compared to Europe. In a study on the Norwegian population, the prediction success of red hair color was 97% and black hair was 93%, while the prediction success of brown was 70% and blond hair was 72% [29]. Our findings for black hair (95.23%) color agreed with this study, and we observed a higher prediction success for brown hair (96.90%) color whereas the red hair (75%) and blond hair (59%) colors were lower, as explained above. Blond/brown hair color phenotypes have been reported as challenging in terms of accurate prediction due to their age-dependent changes in hair color. The blond hair phenotypes during childhood often darken over the years. The molecular structure of these changes is still not clear [16,30]. The brown hair color prediction success was lower in other European population studies compared to our results [16,29,31]. Blond hair phenotypes are more common in Europe than in the Turkish population. Therefore, age-related hair color changes may also occur more frequently, resulting in an increased rate of false predictions. In our study population, the blond hair phenotype (14%) was much lower than brown hair (65%). Most of the brown hair-colored individuals were not affected by age-dependent changes in our cohort. Thus, this resulted in high accuracy in brown hair prediction. On the other hand, the blond hair prediction was the most erroneous prediction in our sample set, and 11 individuals with blond hair were assigned as having a brown hair color. Similar incorrect predictions were also observed in other studies [29,32,33]. This may also be explained as a subjective perception of hair color by individuals, and it is sometimes difficult to distinguish dark blond from light brown.

The HIrisPlex model yields additional hair color shade prediction values (light and dark) to aid in the better classification of each hair color. The *p*-value of the light color shade provides additional information for blond hair color predictions [16,29].

The EVCs (e.g., eye and hair colors) are the most visible variations between populations. Individuals from different geographical regions may have different SNP variations that might affect their EVCs. In this study, we observed allele frequency differences in three SNPs (rs683, rs16891982, and rs12913832) between the Turkish and European populations. These allele frequency differences again show how important it is to test the HIrisPlex panel in non-European populations. As discussed above, the prediction performance of the eye and hair colors were reasonable for the blue and brown eye colors, and black and brown hair colors. However, non-European populations have more variation in the intermediate phenotypes compared to Europeans, which resulted in false predictions for those phenotypes. Therefore, the interpretation and reporting should be made more carefully for these phenotypes and potential errors should be addressed. Improvement in the prediction of challenging phenotypes needs further studies for a better understanding of the genetic structure and additional new markers.

The determination of intermediate eye and blond/brown hair colors may be subjective. To eliminate subjectivity, it is necessary to standardize the collection and reporting of EVCs. To increase consistency, the standardization of nomenclature is also required [34]. Moreover, a guideline or standard interpretation form could be added to the case report for a better understanding of the % correct predictions per phenotype as a means of indicating potential errors.

## 4. Conclusions

In this study, we assessed the performance of the HIrisPlex system (SNaPshot method and MLR model) for hair and eye color prediction in the Turkish population. We determined the prediction performance by calculating the sensitivity, specificity, AUC, PPV, and NPV values of the HIrisPlex panel. We obtained high prediction accuracies (over > 95%) for blue and brown eye colors and black and brown hair colors. The prediction errors were observed in the more complex phenotypes such as intermediate eye color and blond hair color. Despite the challenges with these phenotypes, the HIrisPlex system can be reliably applied to the Turkish population when interpreted with caution.

## Figures and Tables

**Table 1:** The classification for each different eye color category and summary statistics (AUC, sensitivity, specificity, PPV, and NPV) using HIrisPlex for the Turkish population.

	Predicted % (Bold Numbers Indicates Prediction Success Percentage)					Summary Statistics				
HIrisPlex (Eye Color)	Blue	Brown	Intermediate	Undetected	Samples	AUC ^a^	Sensitivity %	Specificity %	PPV % ^b^	NPV % ^c^
**Blue**	**100**	0	ND	0	20	0.66	100	92.24	66.66	100
**Brown**	0	**95.60**	ND	4.39	91	0.88	95.60	65.51	81.30	90.47
**Intermediate**	26.31	52.63	ND *	21.05	38	0.59	0	100	ND	74.49

* ND: Not Determined; ^a^ Area under the curve; ^b^ Positive predictive value; ^c^ Negative predictive value.

**Table 2:** The classification for each different hair color category and summary statistics (AUC, sensitivity, specificity, PPV, and NPV) using HIrisPlex for the Turkish population.

	Predicted % (Bold Numbers Indicates Prediction Success Percentage)					Summary Statistics %				
HIrisPlex (Hair Color)	Black	Brown	Blond	Red	Samples	AUC ^a^	Sensitivity %	Specificity %	PPV % ^b^	NPV % ^c^
**Black**	**95.23**	4.76	0	0	21	0.96	95.23	98.43	90.90	99.21
**Brown**	2.06	**96.90**	1.03	0	97	0.89	96.90	76.92	88.67	93.02
**Blond**	0	40.74	**59.25**	0	27	0.90	59.25	98.36	88.88	91.60
**Red**	0	0	25.00	**75.00**	4	0.55	75.00	100	100	99.31

^a^ Area under the curve; ^b^ Positive predictive value; ^c^ Negative predictive value.

## Data Availability

The data that support the findings of this study are available from the corresponding author upon reasonable request.

## Supplementary Materials

The following supporting information can be downloaded at: https://www.mdpi.com/article/10.3390/genes13112094/s1, Table S1: Allele frequencies of the Turkish and European population from 1000 Genomes Project Phase 3 data.

## Abbreviations

EVCs	Externally visible characteristics
FDP	Forensic DNA phenotyping
MLR	Multinomial logistic regression
PISNPs	Phenotype informative SNPs
SBE	Single-base extension
